# Identification of a Low Digestibility δ-Conglutin in Yellow Lupin (*Lupinus luteus* L.) Seed Meal for Atlantic Salmon (*Salmo salar* L.) by Coupling 2D-PAGE and Mass Spectrometry

**DOI:** 10.1371/journal.pone.0080369

**Published:** 2013-11-22

**Authors:** Takahiro Ogura, Adrián Hernández, Tomoko Aizawa, Jun Ogihara, Michio Sunairi, Javier Alcaino, Haroldo Salvo-Garrido, Iván J. Maureira-Butler

**Affiliations:** 1 Genomics and Bioinformatics Unit, Agriaquaculture Nutritional Genomic Center (CGNA), Temuco, IX Región, Chile; 2 Núcleo de Investigación en Producción Alimentaria/Escuela de Acuicultura, Facultad de Recursos Naturales, Universidad Católica de Temuco, Temuco, IX Región, Chile; 3 College of Bioresource Sciences, Nihon University, Fujisawa, Kanagawa, Japan; 4 Institute of Agricultural Research (INIA Carillanca), Temuco, IX Región, Chile; University of Strathclyde, United Kingdom

## Abstract

The need of quality protein in the aquaculture sector has forced the incorporation of alternative plant proteins into feeding diets. However, most plant proteins show lower digestibility levels than fish meal proteins, especially in carnivorous fishes. Manipulation of protein content by plant breeding can improve the digestibility rate of plant proteins in fish, but the identification of low digestibility proteins is essential. A reduction of low digestibility proteins will not only increase feed efficiency, but also reduce water pollution. Little is known about specific digestible protein profiles and/or molecular identification of more bioavailable plant proteins in fish diets. In this study, we identified low digestibility *L. luteus* seed proteins using Atlantic salmon (*Salmo salar*) crude digestive enzymes in an *in vitro* assay. Low digestibility proteins were identified by comparing SDS-PAGE banding profiles of digested and non-digested lupin seed proteins. Gel image analysis detected a major 12 kDa protein band in both lupin meal and protein isolate digested products. The 12 kDa was confirmed by 2D-PAGE gels and the extracted protein was analyzed with an ion trap mass spectrometer in tandem mass mode. The MS/MS data showed that the 12 kDa low digestibility protein was a large chain δconglutin, a common seed storage protein of yellow lupin. Comparison of the protein band profiles between lupin meal and protein isolates showed that the isolatation process did not affect the low digestibility of the 12 kDa protein.

## Introduction

Protein is a major component in most fish diets. Historically, fish meal has been the main protein source for most of the aquaculture industry [1]. However, the increasing demand of aquafeeds and the underperfomance of several fisheries have pushed fish meal prices to the point of threatening or restraining growth of the aquaculture sector [2], [3]. Thus, a number of efforts have been carried out to find alternative protein sources of high nutritional quality and readily bioavailable for aquafeeds [4], [5].

The substitution of fishmeal with lupin meal in diets for salmonid species has been reported with satisfactory results in terms of growth and digestibility by various authors [6], [7], [8], [9], [10]. Among domesticated lupins, *L. luteus* shows higher protein seed content and digestibility than other lupin species [8] and twice the amount of seed cysteine and methionine, two essential aminoacids commonly deficient in plant proteins [9]. The main lupin seed proteins are storage proteins, and were initially classified based on their electrophoretic mobility as α-, β-, γ-, and δ-conglutins [11]. However, subsequent protein separation studies that were more technologically advanced suggested significant protein fraction heterogeneity [12], [13]. Recent proteomic studies, carried out by combining 2D electrophoresis and mass spectrometry, have produced specific aminoacidic sequences, which have allowed the identification and classification of most storage proteins within each main conglutin group in *L. albus* and *L. angustifolius* [14], [15], [16].

Although digestibility of lupin seed proteins is sufficient for their utilization as a feed ingredient, a fraction of its protein has low digestibility levels [9], [17], reducing feed efficiency and increasing the odds of nitrogen water pollution. The presence of protease inhibitors has been associated with the reduction of protein digestibility; however, the lupine protease levels are only 5% of soybean meal [18]. Anti-nutritionals, such as fibers and oligosaccharides, have also shown negative effects on protein digestibility [19], [20]. Feeding studies using protein concentrates and isolates, where oligosaccharides and fibers were mostly eliminated, have shown significant increases in organic matter, energy and protein digestibility [21]. Efforts to increase digestibility have been also carried out using exogenous commercial enzymes with moderate, positive effects [22]. *In vitro* results using ruminal fluid and conglutin fractioning studies have suggested that some lupin conglutins are more digestible than others [17], [11]. In addition, differences in digestibility have also been observed among lupin cultivars, where the amount of identifiable protein varied after being digested by ruminal fluid extracts [17]. Although recent efforts have focused on studying the genetic molecular diversity contained within *L. luteus* [23], [24] its seed proteome or the specific digestibility level of its storage proteins have received little attention.

The purpose of this study was to detect and identify low digestible *L. luteus* seed proteins under an *in vitro* assay using salmon digestive crude extracts. Proteins were extracted from de-hulled seed meal, and sequentially digested by salmon stomach and pyloric caeca extract; and thus, mimicking the salmon digestive track. Detection and identification of low digestible proteins was carried out by coupling 2D-PAGE electrophoresis and mass spectrometry, providing insights into the yellow lupin proteins that will be the future targets of breeding efforts to increase feed efficiency.

## Materials and Methods

### Chemicals

Azocasein and azoalbumin were purchased from Sigma-Aldrich Co. LLC (MO, USA). Protein molecular weight marker (Mark 12™ Unstained Standard) was purchased from Life Technologies Co. (NY, USA). Carrier ampholytes (Pharmalyte™ pH 3–10 for IEF [isoelectric focusing] and Ampholine™ pH 4.5–6.5 for IEF) were purchased from GE Healthcare Bioscience (PA, USA). Skim milk (CALO®; Watt's S.A., Osorno, Chile) was purchased at a local market. Deionized water was used in this study. All other reagents used in this study were of ACS reagent, molecular biology, or electrophoresis grade.

### Preparation of Dehulled Seed meal and Protein Isolate

Seeds of a sweet yellow lupin cultivar (*Lupinus luteus* cv. Wodjil) were harvested from a variety trail located at the CGNA's experimental fields, INIA Carillanca, Temuco, Chile (lat 38°41′S, long 72°25′W) in 2009. Seeds were partially crushed and seed coats manually removed. Dehulled seeds were milled by a grinding mill, and the meal was passed through a 106 µm sieve. Lupin protein isolates were made from this meal. All following procedures were conducted at room temperature. Meal was defatted three times by five volumes (v/w) of n-hexane, and air dried. Protein isolates were prepared by a two step process [25]. In the first step, protein was extracted from the defatted meal using five volumes (v/w) of water for 1 h at pH 4.5 and clarified by centrifuging at 8,000× g for 10 min (crude fraction F). In the second step, protein from the precipitated residue previously obtained was extracted using 10 volumes (v/w of initial defatted meal) of water for 1 h at pH 7.2. This extract (crude fraction E1) was clarified by centrifuging at 8,000× g for 10 min. The precipitated residue was extracted again at pH 7.2 with five volumes (v/w of initial defatted meal) of water, to produce the crude fraction E2. Crude fractions E1 and E2 were mixed, and their protein precipitated at pH 4.5, overnight. Most of the supernatant was removed by decantation, and the precipitated protein was further concentrated by centrifuging at 8,000× g for 10 min and then freeze dried (fraction E). Meanwhile, the pH of the crude fraction F was adjusted to 8.0 and left to rest overnight. This crude fraction F was clarified by centrifuging at 8,000× g for 10 min and concentrated to one-tenth volume by ultrafiltration using a six unit tandem connected Vivaflow 50 (10,000 MWCO PES; Sartorius Stedim Biotech GmbH, Goettingen, Germany). This concentrated crude fraction F was diafiltrated by two volumes (v/v of the concentrated fraction F) of water with the same device. Protein from this diafiltrated fraction F was precipitated with five volumes (v/v of diafiltrated fraction F) of ethanol. The precipitated protein (fraction F) was dried at 60°C. Finally, dried fractions E and F were mixed. Protein levels of this isolates were 96.7% (calculated from the determination of total nitrogen by Kjeldahl digestion, based on N x 6.25).

### Casein preparation

Casein was prepared from dry skim milk. Skim milk was dissolved in 10 volumes (v/w) of water. Protein from this solution was precipitated at pH 4.5, and concentrated by centrifuging at 8,000× g for 10 min at room temperature. The precipitated protein was stored at 4°C until use.

### *In Vitro* Digestion Assay Substrates Preparation

The substrate protein was extracted from the dehulled seed meal, protein isolates, and casein. Each material was mixed with 10 vol (v/w) of water for 90 min at pH 2, room temperature. The extracts were clarified by centrifuging at 9,000× g for 10 min at ambient temperature. Protein content was quantified as described by Bradford [26] and using bovine serum albumin as a standard. The extracts were stored at −20°C until use.

### Salmon Crude Digestive Enzyme Preparation

The stomachs and pyloric caeca were collected from 4 d fasted Salmon (*Salmo salar*; average weight 50 g) to prepare samples of digestive enzymes. All groups of fish were placed in chilled water and anesthetized with isoeugenol (Aqui-s®; Bayer Animal Health, Santiago, Chile) prior digestive extractions. All animal experimental procedures and protocols were performed in accordance with current Chilean regulations and with the approval of the Bioethics Committee of the Catholic University of Temuco. The stomachs were homogenized using an ice-cold mortar and mixed with four volumes (v/w) of water for 4 h on ice. This extract was clarified by centrifuging at 9,000× g for 10 min at 4°C, and the extract allowed to rest over night at 4°C. The extract was further clarified by centrifuging at 9,000× g for 10 min at 4°C, and stored at −80°C until use. The pyloric caeca was extracted as described by Yoshimura *et al.* [27]. Briefly, pyloric caeca were homogenized using an ice-cold mortar and mixed with three volumes (v/w) of a 1 mM CaCl_2_ cold water solution. The pH was adjusted to 5.0, and the mixture let rest on ice for 15 min. The extract was clarified by centrifuging at 9,000× g for 10 min at 4°C. The pH of this extract was adjusted to 8.0, and left to rest over night at 4°C. The extract was further clarified by centrifuging at 9,000× g for 10 min at 4°C, and stored at −80°C until use. Protease activities of the each extract were measured as described by Charney and Tomarelli [28] using 1.25% (w/v) azoalbumin (pH 2.0) for the stomach extracts and 1.25% (w/v) azocasein (pH 8.0) for the pyloric caecum extracts, respectively. One unit of protease activity was defined as a volume of the extract that raises absorbance for 0.1 at 440 nm for 1 h at 30°C.

### *In Vitro* Digestion Assay

A 12.5 µL aliquot of a substrate solution containing 20 µg of protein in 60 mM glycine (pH 2.0) were pre-incubated for 15 min at 15°C. After the pre-incubation, 2.5 µL (0.032 U) of the stomach extract were added to the solution, and incubated for 3 h at 15°C. Next, 3 µL of the alkaline buffer consisting of X mM NaOH (where X was preliminary determined for each substrate to reach pH 8 of the test solution), 333 mM Tris-HCl (pH 8.0), and 6.67 mM CaCl_2_ were mixed with the solution, and incubated for 15 min at 15°C. Next, 2 µL (0.56 U) of the pyloric caecum extract were added to the solution, and incubated for 3 h at 15°C. After incubation, the solution was immediately put on a pre-chilled tube container, and the digested product chilled at −80°C. Digestion assays without the crude digestive enzymes (defined as a non-digested product in the results section) and without substrate (defined as a blank in the results section) were also conducted as controls following the same procedure. The chilled digested products were separated on SDS-PAGE as follows. Five micro liters of 6× SDS sample buffer consisting of 0.375 M Tris-HCl (pH 6.8), 150 mM DL-dithiothreitol (DTT), 12% sodium dodecyl sulfate (SDS), 30% sucrose, and 0.012% bromophenol blue were loaded in the inner wall of the test tube near the chilled digested product, and this tube was immediately heated for 5 min at 100°C. Proteins in the digested products were separated by SDS-PAGE (sodium dodecyl sulfate polyacrylamide gel electrophoresis) with Tris-tricine buffer [29] on a 12% acrylamide gel (10 cm ×10 cm). Proteins were stained using coomassie brilliant blue G-250 procedure, as described by Neuhoff *et al.* [30], and the gel image digitized using a LAS 3000 imaging system (Fujifilm, Tokyo, Japan). The whole intensity of each gel lane was used to estimate digestibility. Lane intensity was quantified using the Analyze Gel function of ImageJ 1.46e (Wayne Rasband, National Institute of Health, USA) and protein digestibility calculated as follows:

Digestibility (%)  = 100×{1−(D−B)/(N−E)}.

D, the whole intensity of a digested product lane.

B, the whole intensity of a blank lane.

N, the whole intensity of a non-digested product lane.

E, whole intensity of a vacant lane.

All digestion assays were repeated three times for each substrate. Student's *t*-tests were conducted to compare protein digestibility among substrates. Protein digestibility was shown at an average ± standard error.

### Identification of low digestibility Proteins

An *in vitro* digestion assay was carried out to identify yellow lupin proteins with low digestibility levels. Ninety micrograms of proteins that were extracted from dehulled seed meal were applied on the digestion assay, as described above (total volume 57 µL), and proteins in the digested product were precipitated by adding 60 µL of 20% trichloroacetic acid (TCA) and 1.2 mL of ice-cold acetone. The precipitate was washed two times using 80% and 100% acetone, respectively, and later air dried. Proteins were separated by two-dimensional polyacrylamide gel electrophoresis (2D-PAGE) as described by O'Farrell (1975) with some modifications. First, proteins were dissolved in an isoelectric focusing (IEF) solution consisting of 7 M urea, 2 M thiourea, 4% 3-[(3-Cholamidopropyl) dimethylammonio]-1-propanesulfonate hydrate (CHAPS), 65 mM DTT, and 2% carrier ampholytes (pH 3.5–10∶pH 4–6.5 = 1∶4). The IEF/protein solution was loaded into a Mini-PROTEAN® 2-D Electrophoresis Cell (Bio-Rad, CA, USA) for 3.5 h at 750 V according to manufacturer's specifications. Then, the protein in the tube gel was fixed using 20% TCA for three minutes, washed with distilled water for 2 h to remove the carrier ampholytes, and stored at −80°C until use. Later, the gel was equilibrated in a solution consisting of 50 mM Tris-HCl (pH 8.8), 6 M urea, 30% (w/v) glycerol, 2% SDS, 1% DTT, and an aliquot of bromophenol blue for 10 min. Finally, proteins were further separated in a 12% SDS-PAGE gel using a Tris-tricine buffer, and visualized by staining with a coomassie brilliant blue G-250 solution as described above. 2D PAGE electrophoresis protein separation was conducted tree times, and only proteins that consistently were visualized in all three gels were considered to have low digestibility. The crude enzymatic digestive extract was also put through 2D PAGE electrophoresis and used as a blank control.

Low digestibility proteins were analyzed and identified using a mass spectrometer, as previously described by Mori et al. [31]. In brief, proteins (spots) were excised from the gel, destained, and the thiol groups of cysteines alkylated by iodoacetamide. Proteins were digested using trypsin, and peptides analyzed with liquid chromatography coupled with an ion trap mass spectrometry, in a tandem mass mode. The obtained tandem mass spectrometry (MS/MS) data were analyzed using the software Mascot MS/MS Ion Search, web version (Matrix Science LTD., London, UK) under the following parameters: databases, NCBInr 20121021 and contaminants 20090624; enzyme, trypsin; fixed modification, carbamidomethyl; variable modification, oxidation (M); mass value, average; peptide mass tolerance, ±2 Da; fragment mass tolerance, ±0.8 Da; missed cleavage, 1; instrument, ESI-TRAP. Searching results were considered as a true positive when at least two MS/MS peptide data were matched to a common protein in the database at p<0.05.

## Results

### SDS-PAGE protein profiles and detection of low digestibility Proteins

**Figure 1:**
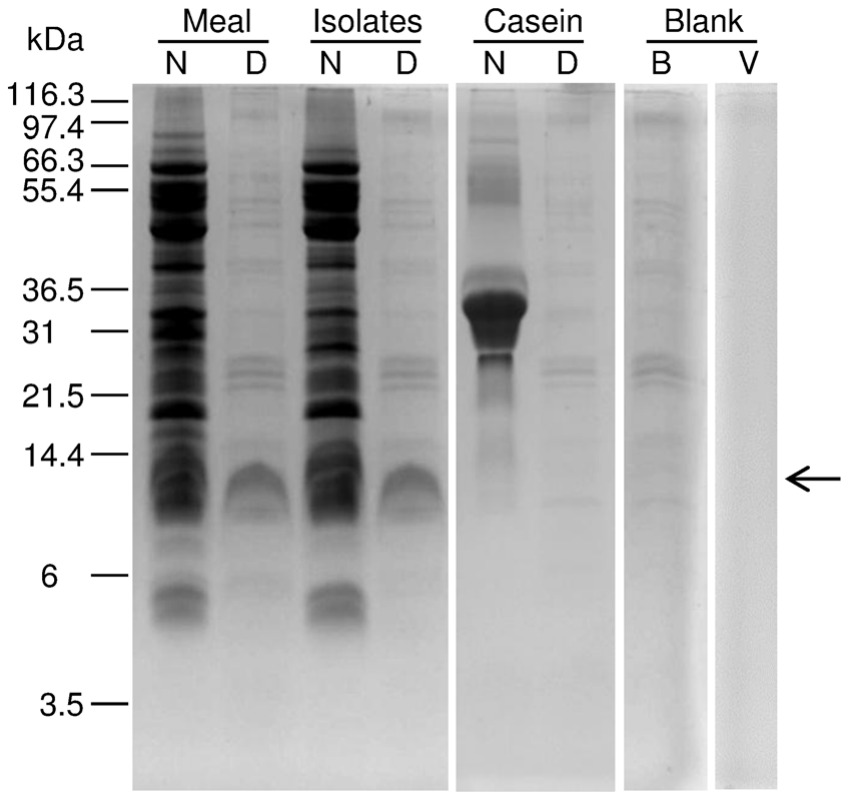
Protein band profiles of non-digested and digested yellow lupin products. Meal, the dehulled seed meal extracts; Isolate, the protein isolate extracts; Casein, casein extracts; N, non-digested product; D, digested product; B Blank, only digestive enzymes; E, Blank, empty lane. The position of the main low digestibility lupin protein is shown by a leftward-pointing arrow.

Protein *in vitro* digestion assays were conducted to detect low digestibility seed lupin proteins. SDS-PAGE profile comparisons among digested and non-digested protein products showed that the salmon enzymatic crude was able to digest both seed meal and isolate lupin proteins (Figure 1). In addition, comparisons between digested and non digested casein (positive control) banding profiles showed that the salmon digestive crude was fully active during the *in vitro* assay.

**Figure 2:**
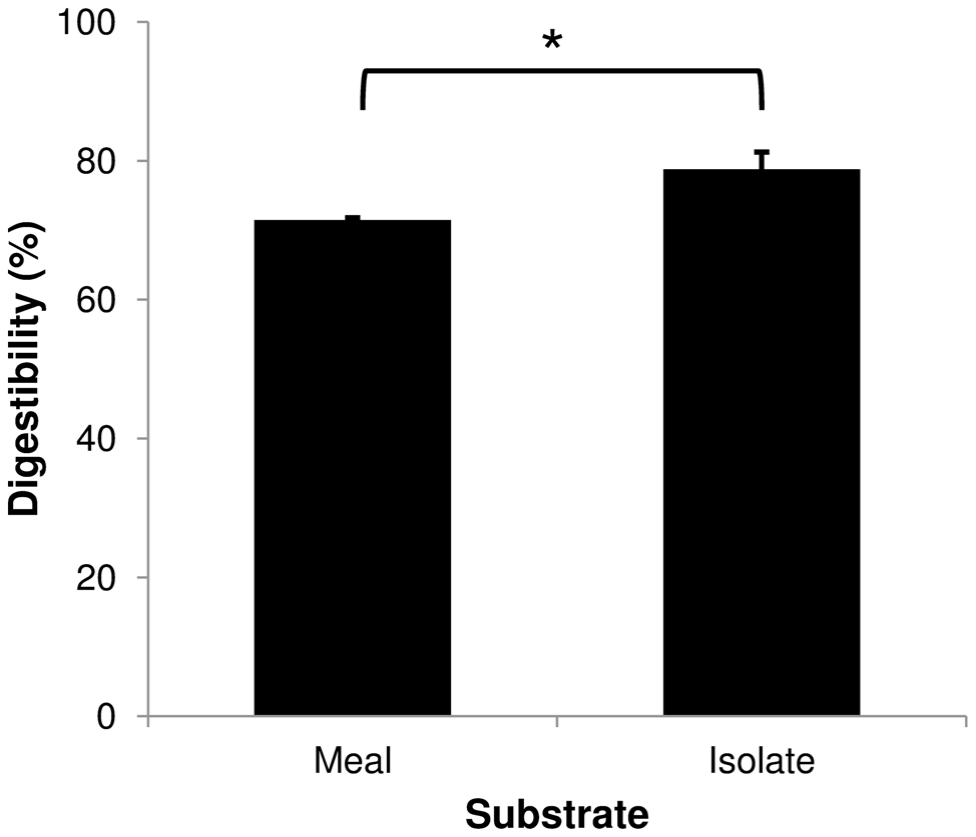
Protein digestibility for yellow lupin meal and protein isolate. Meal, the dehulled seed meal extracts; Isolate, the protein isolate extracts. (*) significantly different (p = 0.043).

A more detailed visual inspection of yellow protein band profiles showed that at least four bands of 88-, 33-, 31-, and 17-kDa were observed clearly on the non-digested meal lane but not detected, or significantly reduced, on the non-digested isolate lane (Figure 1). No evident differences were observed between digested (meal vs. isolate) yellow lupin protein profiles. A unique and strong 12KDa band was detected in both digested lupin substrates (Figure 1). Other weaker bands were also observed; however, their presence in the blank and positive controls suggested that these proteins were most likely of fish origin (salmon digestive extracts). Protein *in vitro* digestibility was calculated by comparing whole band intensity from gel images between non-digested and digested lupin substrates. A significant but small digestibility difference (p = 0.043) was observed between lupin substrates which varied between 71.47% and 78.80% for lupin meal and isolate, respectively (Figure 2).

### Identification of low digestibility Proteins

**Figure 3:**
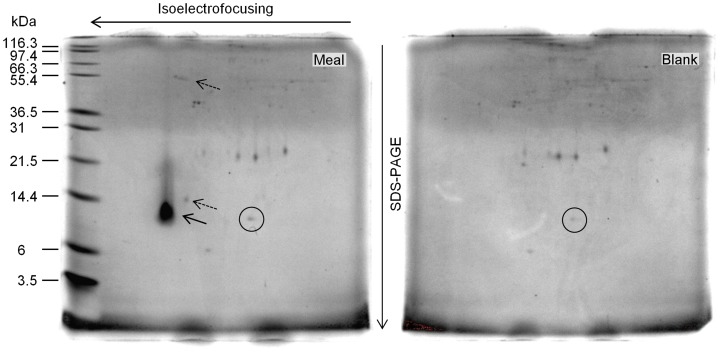
2D-PAGE gel images of yellow lupin digested products. Meal, the dehulled seed meal extracts; Blank, only digestive enzymes. The leftward-pointing arrow and the leftward-pointing dotted arrow point the 12 kDa and other minor indigestible yellow lupin proteins, respectively. The circle shows a protein from the digestive enzymes.

To identify the low digestibility 12 kDa protein(s) observed on the SDS-PAGE profiles, a second *in vitro* digestibility assay was conducted using lupin meal as substrate. The digested products were separated using 2D-PAGE gels, and protein spots were visualized by staining with coomassie brilliant blue G-250. Figure 3 shows 2D-PAGE gel images of the digested meal products and the blank control containing only the digestive crude extract. As in the SDS-PAGE gels, a clear 12 kDa protein was observed in the digested meal product. One weak spot was observed at 14 kDa and a group of faint spots were detected at 55 kDa. None of the 12, 14 y 55 KDa spots were observed in the blank gel (digestive crude extract).

**Figure 4:**
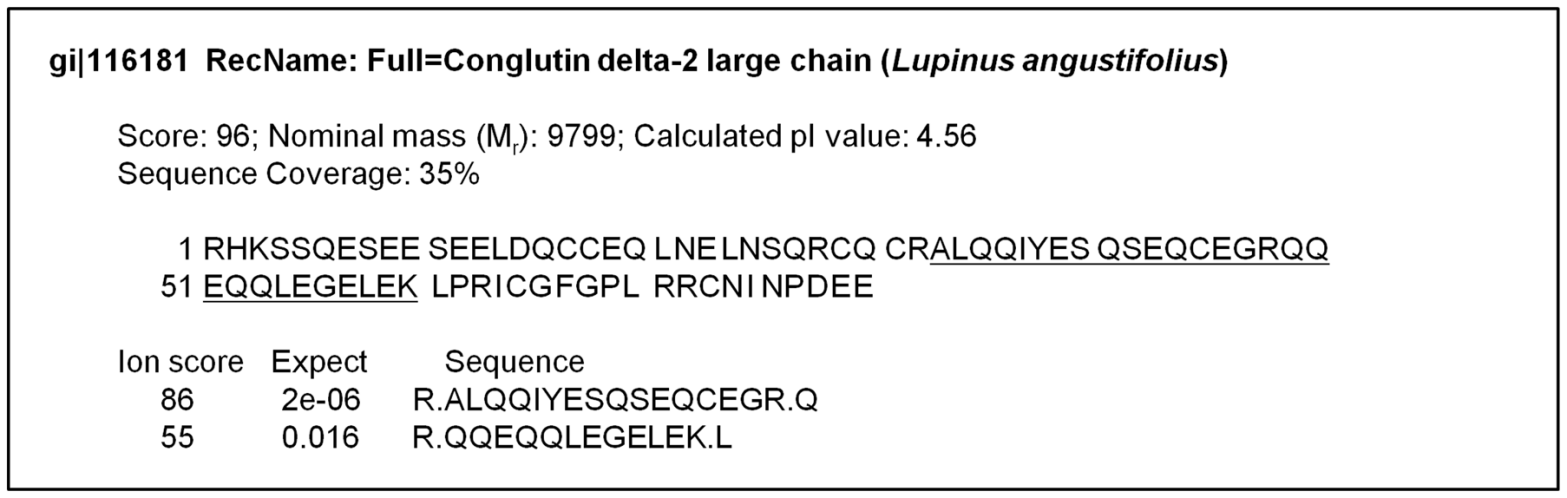
The identified 12 Partially isolated 12-PAGE was excised and analyzed with liquid chromatography coupled to ion trap mass spectrometry in a tandem mass mode. The identified peptides in the amino acid sequence are underlined.

The main low digestibility protein (12 kDa) spot was excised; trypsin digested and analyzed using liquid chromatography coupled to an ion trap mass spectrometer in a tandem mass mode. The obtained peptide MS/MS data were analyzed against the NCBInr and contaminants data base. Two peptides matched the conglutin δ-2 large chain of the narrow-leaved blue lupin (*Lupinus angustifolius*; gi|116181; Fig. 4), strongly suggesting that the 12 KDa *L. luteus* low digestibility protein was a conglutin delta storage protein.

## Discussion

Protein digestibility has been studied in a number of species, using both *in vivo* and *in vitro* assays [32], [4], [17], [33]. Although *in vivo* experiments provide more realistic and accurate digestibility estimations [34], [35], *in vitro* assays have proven to be a valid alternative, especially when costs, experimental time length, availability of raw ingredients, and management of live test organisms are an issue [35]. In addition, *in vitro* digestibility assays have allowed answering specific questions given the reduction of uncontrolled factors associated to live animal test systems. For instance, protein digestibility is affected by presence of non-protein compounds, such as oligosaccharides, in the tested samples [19], and some soluble proteins are missed during feces collection due to water dispersal in fish studies [36]. Our *in vitro* assays allowed us to detect and identify yellow lupin seed proteins with low levels of digestibility when exposed to salmon digestive crude. *In vitro* digestibility comparisons between lupin meal and isolate showed a small, but significant (*p* = 0.043) difference between lupin substrates (Figure 2). Plant compounds previously reported to affect protein digestibility, such as α-galactosides and phytic acid [19], [37], were eliminated when generating the protein isolate. Thus, digestibility differences could be explained, at least in part, by the presence/absence of these compounds in the lupin meal and isolate. For instance, Sajjadi and Carter [37] found phytates reduced protein digestibility in Atlantic salmon, but had no effect on feed intake, growth or trypsin activity. Studies in rainbow trout showed that degradation or extraction of α-galactosides by adding α-galactosidase or ethanol extracting processes, respectively, did improve nitrogen and organic matter digestibility [19].

SDS-PAGE gel visual comparisons showed that both non-digested lupin substrates banding profiles were similar (Figure 1). Few (4) extra proteins were observed in the non-digested lupin meal but not in the non-digested isolate, suggesting protein break down, or some type of selection during the isolate generation process. In the case of digested substrates, a common clear and unique 12 kDa protein band was observed in both lupin meal and isolate (Figure 1). 2D SDS-PAGE gels confirmed the existence of protein digestibility differences, being the 12 KDa protein the major one (Figure 3) and the obvious candidate for protein identification.

Peptide MS/MS datastrongly suggested our low digestibility 12 KDa protein corresponded to the large chain of a conglutin δ (Figure 4). Conglutin δ belongs to a 2S seed storage lupin protein class, which consists of two subunits (namely large and small chain). The subunits are chained by two disulfide bonds, and the large subunit has two extra intrachain disulfide bonds [38], which have shown negative effects on protein digestibility [39], [40], [41]. For instance, Oria et al. [40] found that decrease in digestibility was due, at least in part, to the formation of disulfide-bound complexes involving β- and γ-kafirins in maturing sorghum grain.

*In vitro* results obtained using cow ruminal fluid suggested that a portion of *Lupin spp* conglutins α and β were not digested after 24 h of incubation, although the conglutin α sub-fraction was less digestible than conlgutin β [17]. These differences are in agreement with early conglutin fractioning studies, where subfractions of conglutin α showed different levels of solubility [11]. Our study was carried out using a crude salmon digestive sample which has a different digestive enzymatic capability than cow ruminal fluid; clearly pointing out that different animal digestive environments will determine which protein is more or less digestible.

Although our results must be validated in an *in vivo* digestive assay, the information obtained in this study enables us to adopt immunodetection methods to detect conglutin δ from several different yellow lupin meals and excreted feces generated by their consumption. The results in this study will contribute to supply preliminary information to breed yellow lupin cultivars with increased protein bioavailability for aquaculture feeds.
